# Maternal Death: Retrospective Autopsy Study in Southwestern Colombia, 2000–2023

**DOI:** 10.3390/ijerph22071105

**Published:** 2025-07-14

**Authors:** Jhoan Sebastian Cruz-Barbosa, Andrés Felipe Valencia-Cardona, Armando Daniel Cortés-Buelvas, Yamil Liscano

**Affiliations:** 1 Grupo de Investigación en Salud Integral (GISI), Departamento Facultad de Salud, Universidad Santiago de Cali, Cali 760035, Colombia; sebastiancruzb@gmail.com (J.S.C.-B.); andres.valencia.cardona@correounivalle.edu.co (A.F.V.-C.); 2 Departamento de Patología, Facultad de Salud, Universidad del Valle, Cali 760043, Colombia; armando.cortez@correounivalle.edu.co

**Keywords:** maternal mortality, clinical autopsies, Cali, indirect causes, direct causes, social determinants

## Abstract

**Background and aim:** The death of a woman while pregnant or within 42 days of delivery, regardless of the cause of death, or even up to one year after the end of the pregnancy, due to causes related to or aggravated by pregnancy remains a critical public health problem globally and in Colombia. While the country shows a general decreasing trend (preliminary Maternal Mortality Ratio 38.6/100,000 live births in 2023), significant regional disparities persist. Understanding precise underlying causes, especially in high-complexity referral centers, is vital. This study describes the sociodemographic and anatomopathological characteristics associated with autopsy-verified maternal mortality cases at a Level-4 hospital in southwestern Colombia (2000–2023). **Methodology:** A descriptive observational retrospective study analyzed 42 maternal mortality cases verified by clinical autopsy (2000–2023) at the Pathology Department of Universidad del Valle, a Level-4 referral center in Cali, Colombia. Cases met the WHO definition. Data on sociodemographic, clinical, and pathological characteristics were retrospectively extracted from clinical records and autopsy reports. **Results:** The analysis of 42 autopsies (2000–2023) showed that 85.7% were early maternal deaths. Indirect causes predominated (57.1%, n = 24) over direct (42.9%, n = 18). Septic shock was the main indirect cause (65.2% of indirect), often from endemic infections. Hypovolemic shock due to PPH was the main direct cause (50% of direct). A high proportion were from subsidized/uninsured schemes (65.7%) and had a migratory history (20%). **Discussion and conclusions:** This study highlights the value of autopsy in revealing maternal mortality etiologies, showing a predominance of indirect/infectious causes and endemic diseases often missed clinically, despite PPH remaining the main direct cause. Findings reaffirm the strong link between maternal death and social/economic inequity, access barriers, and regional/migratory vulnerabilities. Effectively reducing maternal mortality necessitates rigorous clinical management, regionalized public health strategies addressing inequities, and integrating pathological data for targeted surveillance.

## 1. Introduction

Colombia is a tropical country bordering both the Atlantic and Pacific Oceans. Near the Pacific coast is Cali, the country’s third-largest city and the capital of the Valle del Cauca department. It is located in the Cauca River basin, approximately 900 m above sea level, bordering the Western Cordillera of the Andes mountain range. According to data from the 2018 census and DANE projections, the current population is 2.3 million, a quarter of whom are Black.

Maternal mortality persists as a critical global health challenge, disproportionately affecting low-income nations [1]. Despite a significant 38% reduction over the past decade, the scale remains immense, with more than 800 women dying daily from pregnancy-related causes [2]. While the global maternal mortality ratio (MMR) decreased substantially from 430 per 100,000 live births (LB) in 1990 to 211 in 2017 [3], recent years have seen progress stall, and some areas, like Kampala, Uganda, have even reported sharp increases [4]. This underscores the complexity of the issue, where factors ranging from a country’s Human Development Index to specific causes like hemorrhage, hypertensive disorders, sepsis, and abortion complications play crucial roles [1,2]. Consequently, strengthening health systems, ensuring access to emergency obstetric care, and tackling healthcare inequalities are paramount for renewed progress [4].

Shifting the focus to the national context, Colombia has witnessed a general decline in maternal mortality; however, significant challenges persist, particularly in rural areas and regions affected by conflict. Socioeconomic disparities exacerbate the problem, with mortality rates in the poorest municipalities being nearly three times higher than in affluent ones [5]. Furthermore, the COVID-19 pandemic temporarily reversed progress, causing maternal deaths in 2020 to exceed expected levels by 12.6% [6]. In response to these challenges, interventions such as telehealth and educational programs have been implemented, yielding positive results like reduced obstetric emergencies and improved care access, especially in rural settings [6,7]. Despite these successes, areas impacted by conflict continue to struggle with limited healthcare availability, resource shortages, and geographical barriers [5]. Within this complex landscape, particularly in southwestern Colombia, high-complexity referral centers like the Level-4 university hospital in Cali play a vital role in managing severe obstetric cases, many of which contribute to maternal mortality statistics [6].

Understanding the nature of these severe cases requires recognizing that most complications leading to maternal death are preventable or treatable. These deaths are broadly classified into direct causes, arising from obstetric complications of pregnancy, labor, or postpartum, and indirect causes, stemming from pre-existing conditions or diseases developing during pregnancy that are aggravated by it [8]. Globally, direct causes like hemorrhages (especially postpartum), infections, hypertensive disorders, delivery complications, and unsafe abortions are responsible for the vast majority, around 75% of maternal deaths [2,9,10]. Compounding these clinical factors are social determinants, including income, education, healthcare access, race, and ethnicity, which significantly heighten the risk for vulnerable populations [11].

Specifically within Colombia, recent data reflects these global patterns while providing a current national snapshot. Preliminary figures for 2023 indicate that direct causes accounted for 67.4% of maternal deaths. Consistent with global trends, the leading direct causes were hypertensive disorders associated with pregnancy (19.6%) and obstetric hemorrhage (16.5%), followed by thromboembolic events (9.8%) [12]. These figures emerge after a period where the COVID-19 pandemic temporarily increased the MMR in 2020 and 2021, interrupting a longer-term statistically significant decline observed from 2007 to 2023. The preliminary overall MMR for 2023 settled at 38.6 deaths per 100,000 LB, yet stark regional disparities persist, evidenced by significantly higher rates in territories like Buenaventura, Vichada, and Chocó [12].

While national statistics provide valuable insights, understanding the specific underlying factors, particularly in high-risk regions like southwestern Colombia, requires more detailed investigation. The existing Colombian literature includes studies combining statistical data with autopsy findings, such as one from Santander [13]. However, being several years old and geographically distinct, it leaves a knowledge gap concerning the current situation in the southwest. Clinical autopsies are fundamental in this context, offering a crucial tool to overcome limitations inherent in data derived solely from clinical records or vital statistics. Autopsies allow for the precise identification of the underlying cause of death, revealing clinically unsuspected conditions and clarifying diagnostic uncertainties—advantages particularly pertinent in complex cases managed at referral centers [6]. Therefore, the present study aims to address the identified gap by describing the sociodemographic and anatomopathological characteristics associated with maternal mortality cases identified through clinical autopsies at a major referral hospital in southwestern Colombia between 2000 and 2023, thereby providing updated, region-specific evidence.

## 2. Materials and Methods

### 2.1. Study Design and Population

This was a descriptive observational retrospective study. The study population included all cases of maternal mortality that were admitted for autopsy at the Department of Pathology of the Universidad del Valle in Cali, Colombia, during the period spanning 1 January 2000 to 31 December 2023. This institution serves as a key referral center for medium- and high-complexity healthcare in southwestern Colombia, covering the departments of Valle del Cauca, Cauca, Chocó, and Nariño, particularly for the publicly insured and uninsured populations.

### 2.2. Case Selection

Cases were identified by reviewing the records of all clinical autopsies performed at the Department of Pathology of the Universidad del Valle within the specified timeframe (2000–2023). Inclusion criteria were defined based on the World Health Organization’s definition of maternal death: any death occurring in a woman during pregnancy or within 42 days after delivery of pregnancy and up to one year after the end of pregnancy, regardless of the duration and location of pregnancy, from any cause related to the pregnancy or aggravated by it or its management, but not from accidental or incidental causes [14].

### 2.3. Data Collection

Data were retrospectively extracted from two primary sources: the clinical records of the deceased women and the corresponding autopsy reports from the Department of Pathology.

The following variables were collected:

- Sociodemographic characteristics: Age, provenance (municipality/department), type of health insurance (public, private, uninsured).
- Clinical characteristics: Number of previous pregnancies, gestational age at death, history of abortion, pre-existing pathological conditions (e.g., sickle cell anemia, systemic lupus erythematosus, nutritional disorders, hypertension), type of delivery (vaginal, cesarean, intrauterine fetal death), level of healthcare received (Level 1, 2, 3, 4), duration of hospitalization (days between admission and death).
- Autopsy findings: Macroscopic and microscopic findings relevant to the cause of death.

### 2.4. Determination of Cause of Death

The cause of death for each case was determined based on a comprehensive review of the clinical history and the detailed findings from the macroscopic and microscopic examination during the autopsy. Causes were classified as direct or indirect maternal deaths according to established definitions [14].

The autopsies were performed by the pathology department, with the same two pathologists throughout the period. All cases meeting the case definition were included.

### 2.5. Statistical Analysis

- Descriptive statistical analyses were performed using Stata statistical software (Version 2019, accessed on January 2025).
- Quantitative variables were assessed for normality using the Shapiro–Wilk test. Variables with a *p*-value > 0.05 were considered normally distributed and were summarized using means and standard deviations. Variables that did not meet the assumption of normality (*p* ≤ 0.05) were presented using medians and interquartile ranges.
- Qualitative variables were described using frequencies and percentages.
- Comparisons between categorical variables were performed using the Chi-square test.
- The geographical distribution of cases by provenance was visualized using a map. A choropleth map was created using the online tool Datawrapper (https://www.datawrapper.de/). The platform was accessed on 20 April 2025.

### 2.6. Ethical Considerations

This research adhered to the national regulations for health research in Colombia, as outlined in Resolution 8430 of 1993 by the Ministry of Health and Social Protection, classifying this study as “research without risk” (Chapter 11). Confidentiality and security of all collected data were guaranteed throughout the study. All autopsies performed had informed consent.

## 3. Results

### 3.1. Overview of Autopsied Cases

During the 24-year study period (2000–2023), a total of 42 cases of maternal mortality admitted to the Department of Pathology at the Universidad del Valle underwent clinical autopsies. The majority of these deaths (n = 36, 85.7%) were classified as early maternal deaths (occurring within 42 days postpartum), while 6 cases (14.3%) were late maternal deaths (occurring after 42 days but within 1 year postpartum).

### 3.2. Sociodemographic and Clinical Characteristics

The sociodemographic and clinical characteristics of the study population are summarized in Table 1. Regarding health insurance status, 65.7% (n = 27) of the cases corresponded to women affiliated with the public or uninsured health regimes.

The age distribution of the deceased women was diverse. The highest proportion of cases, 26.2% (n = 11), occurred in the 26–30 age group, followed by the 31–35 age group, 21.4% (n = 9). Women aged 15–20 years represented 23.8% (n = 10) of the cases, while those aged 36–40 years accounted for 11.9% (n = 5)

Gestational age at the time of death varied, with 40.5% (n = 17) of the deaths occurring between 28 and 36 weeks of gestation (third trimester). A significant proportion, 69% (n = 29), of the women had no reported pre-existing medical conditions. Among those with documented antecedents, the most frequent conditions included sickle cell anemia, systemic lupus erythematosus, HTN, and nutritional disorders (including malnutrition and obesity).

Most women—85% (n = 36)—received medical attention at Level-3 or -4 healthcare facilities. The mean duration of hospitalization from admission to death was 10.9 days (range: 1–97 days). The mean hospitalization duration was notably different between direct and indirect causes of death, with indirect causes associated with a longer mean stay (16.5 days) compared to direct causes (4.2 days).

### 3.3. Temporal Trend of Maternal Mortality

**Figure 1:**
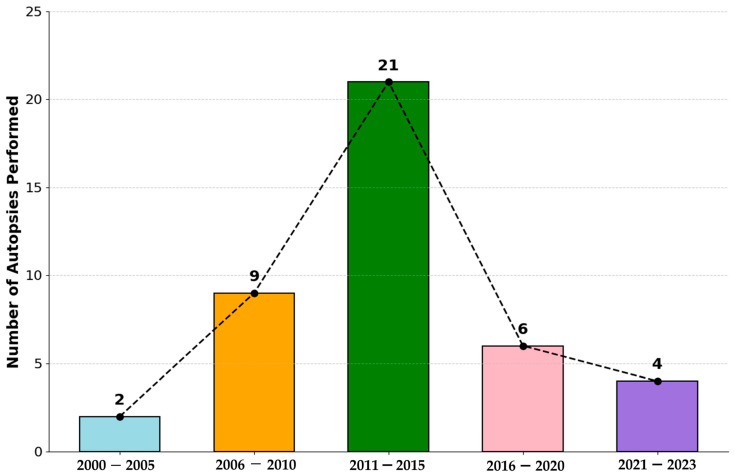
Maternal autopsies performed by five-year periods from 2000 to 2023. A progressive increase in maternal deaths is observed up to the 2011–2015 period, followed by a decline in subsequent periods. Data reflect the total number of registered maternal deaths per period.

Figure 1 illustrates the distribution of maternal autopsies performed quinquennial periods from 2000 to 2023. The data reveal a notable temporal pattern in the number of autopsied maternal deaths. The period spanning 2011–2015 recorded the highest frequency of cases within the analyzed timeframe, with a total of 21 autopsied maternal deaths. Prior to this peak, there was a discernible increase in the number of cases in the preceding quinquennia. Following the 2011–2015 period, a subsequent decline in the number of registered maternal deaths is observed in the later periods presented. It weas important to consider that the temporary suspension of clinical autopsies in Colombia during the COVID-19 pandemic may have impacted the number of autopsied cases registered specifically during that period.

### 3.4. Causes of Maternal Mortality

Causes of death were classified as indirect or direct maternal deaths based on autopsy findings and clinical information.

#### 3.4.1. Indirect Causes of Death

Indirect causes constituted a significant proportion of the recorded maternal deaths, accounting for 24 cases (57.1% of the total). Within this group, the most frequently identified underlying cause was septic shock, present in 65.2% (n = 14) of indirect deaths. Specific etiologies associated with septic shock included bacteremia (which are related to associated events such as pneumonia, urinary tract infections, etc.), pneumonia (multilobar bacterial and cytomegalovirus), massive strongyloidiasis, miliary tuberculosis, and pulmonary tuberculosis, as well as viral meningoencephalitis.

Other shock causes observed among indirect deaths included cardiogenic shock, associated with conditions such as severe mitral valve stenosis with left ventricular dilation and hypertrophic cardiomyopathy secondary to mitral and aortic stenosis due to rheumatic fever. Neurogenic shock was related to extensive ischemic cerebrovascular disease. Hypovolemic shock, combined in some cases with septic shock, was associated with upper gastrointestinal bleeding and abdominal sepsis due to *Escherichia coli*, as well as autoimmune hemolytic anemia. Finally, distributive shock was observed in cases linked to bilateral basal pulmonary hemorrhage, toxic hepatopathy, sickle cell crisis, and metastatic dysgerminoma. The detailed distribution of the number of cases for each specific indirect cause is presented in Table 2.

#### 3.4.2. Direct Causes of Death

Direct causes were responsible for 42.9% (n = 18) of the maternal deaths. Among these, hypovolemic shock due to postpartum hemorrhage (PPH) was the most frequent underlying causes, accounting for a large number—50% (n = 8)—of direct deaths. Specific conditions contributing to hypovolemic shock included uterine atony, hypertensive disorders of pregnancy associated with massive hemorrhagic hepatic necrosis, hemolytic anemia, and placental abruption.

Septic shock also represented a significant direct cause of death, arising from conditions such as panmetritis and septic abortion. Other observed causes among direct deaths included obstructive shock, primarily due to massive pulmonary thromboembolism; cardiogenic or distributive shock, associated with amniotic fluid embolism; and neurogenic shock, linked to cerebrovascular disease in the context of eclampsia, specifically hemorrhagic stroke, as well as fulminant hepatic failure secondary to the acute fatty liver of pregnancy and intracerebral hemorrhage due to arteriovenous malformation. The specific distribution of these direct or obstetric causes and their frequencies is detailed in Table 3.

### 3.5. Geographical Distribution

**Figure 2:**
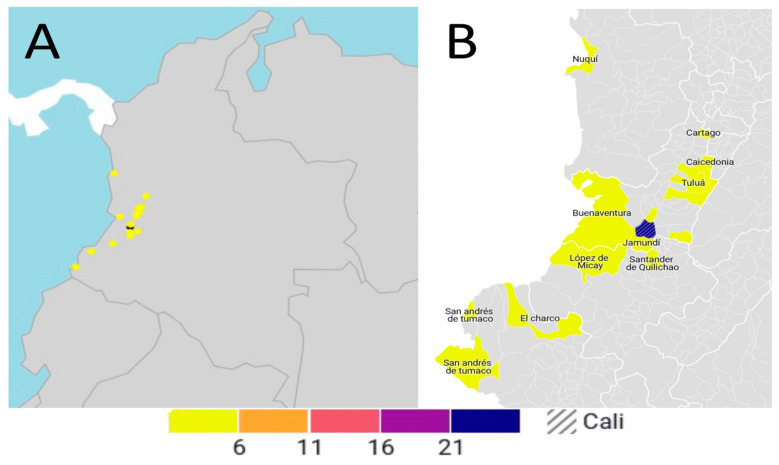
Geographic distribution of maternal mortality for the period 2000–2023. (**A**) Colombian map showing the distribution of maternal mortality. (**B**) Municipalities with at least one maternal death, highlighting Santiago de Cali, the capital of Valle del Cauca, with the highest number of deaths (26), indicated by the hatched pattern. The color scale indicates the number of cases: yellow (6–10), orange (11–15), red (16–20), and purple/blue (21+).

The geographical distribution of maternal mortality cases by provenance across the period 2000–2023 is visually represented in Figure 2. The data indicate a primary concentration of cases originating from Cali, which accounted for the majority at 61.9%. Following Cali, neighboring municipalities within the Valle del Cauca department, specifically those located to the south (e.g., Jamundí), north (e.g., Yumbo), and district of Buenaventura, contributed the next largest proportion of cases. Additionally, cases were registered from patients originating from the departments of Cauca and Chocó. It is relevant to note that these departments are home to significant populations of Indigenous and Afro-Colombian communities, many residing in rural or remote areas. A distinct observation is that 20% (n = 8) of the patients receiving care had a documented migratory background to Cali, with their places of origin identified as the departments of Cauca, Buenaventura, Nariño, and Chocó.

## 4. Discussion

### 4.1. Main Findings

Between 2008 and 2023, approximately 259 maternal deaths were reported in the city of Cali, including traumatic and natural deaths. At the referral center of the University Hospital of Valle, autopsies are performed only for natural causes. It is important to note that the suspension of clinical autopsies during the health emergency may have led to an underreporting of cases in previous years, which affects historical comparability and should be considered when evaluating temporal trends [5].

Beyond the general trends, the analysis of the specific characteristics of the studied cohort reveals important details. For example, the age profile of the deceased patients, with a higher proportion of deaths among those aged 26 to 30 years (26.2%), differs slightly from national data for Colombia, where the 20 to 24 age group is more frequently affected [15]. This regional difference could be influenced by local factors or by the selection bias inherent in autopsy-based studies.

Regarding sociodemographics, a relevant finding is the high proportion of patients belonging to the public or uninsured health scheme (65.7%) in our cohort, although slightly lower than the 90% reported nationally in these groups. This characteristic is critical, as the national and international literature has consistently demonstrated that affiliation with the subsidized scheme or lack of insurance are significant risk factors for maternal mortality (RR 1.90 and 1.57, respectively, in Colombia) [15,16], likely reflecting barriers to timely and quality healthcare access.

### 4.2. Trend Analysis and National Context

There is a marked regional heterogeneity regarding maternal mortality in Colombia, which is often masked by national averages. Departments such as Chocó, for example, exhibit alarmingly high figures, with an MMR of 224/100,000 LB between 2010 and 2018, comparable to that of low-income African countries [17]. In contrast, regions like Bogotá have historically had considerably lower MMRs; however, even in the capital, the study by [18] on early maternal mortality in Bogotá reported a decrease from 39 (2010–2012) to 32 (2013–2015). These geographical disparities and the extreme differences observed even at the municipal level reflect structural inequities linked to socioeconomic factors such as poverty and low health insurance coverage, geographical access barriers, weaknesses in health infrastructure, cultural and ethnic factors, and, in some regions, the impact of armed conflict [17,19,20].

The monitoring of maternal mortality in Colombia is fundamentally based on national surveillance systems such as the National Public Health Surveillance System (SIV-IGILA) and DANE vital statistics. The quality and completeness of these systems are essential for obtaining reliable MMR estimates and understanding their causes. Despite their importance, inherent limitations exist, such as the potential underreporting of cases or the incorrect classification of causes of death. For example, the temporary suspension of clinical autopsies during the COVID-19 pandemic may have affected historical comparability and generated underreporting [5]. To improve data quality, confrontation with vital statistics is used and, in some contexts, complementary methods such as clinical autopsy are employed, as was performed in the base study in Cali and in other regional studies [13]. The interpretation of MMR trends, therefore, must consider these methodological and contextual complexities, especially when comparing periods affected by extraordinary events such as the pandemic.

### 4.3. Comparative Analysis of the Causes of Maternal Death

An analysis of various studies in Colombia reveals varied epidemiological profiles regarding the causes of maternal death (see Table 4). Nationally, epidemiological surveillance data often point to Hypertensive Disorders in Pregnancy (HDP) and hemorrhagic complications as the main direct causes [21,22]. The autopsy study in Cali (current study) shows a predominance of indirect causes (57.1%), with septic shock as the main pathophysiological endemism (65.2% of indirect causes), often derived from non-obstetric infections. In contrast, direct causes in Cali (42.9%) were led by hypovolemic shock secondary to PPH (50% of direct causes).

Comparison with other regional studies shows diverse profiles. For example, an autopsy-based study in Santander also reported a predominance of indirect causes (67.8%), with non-gynecobstetric infections as the most relevant cause (45.2% of indirect causes) [13]. In Bolívar, a clear predominance of direct causes was found (62.7%), led by obstetric hemorrhage [21]. The analysis in Antioquia, on the other hand, centered on HDP as the main cause of death [22]. This interregional variability underscores the importance of local analyses and autopsy-based studies for understanding the specific epidemiological profiles of each territory and adapting prevention strategies, further highlighting the persistence of PPH as a crucial direct cause both nationally and globally [24].

### 4.4. Comparative Analysis of Sociodemographic and Clinical Profiles

The profile of women who die from maternal causes presents recurring characteristics in various studies (see Table 4). The autopsy study in Cali (current study) found that the largest proportion of deaths occurred in the 26–30 age group (26.2%). While this varies slightly from national reports that often point to younger groups such as 20–24 or 25–29 years as having the highest concentration of cases [15], the risk of maternal mortality is consistently higher at the extremes of reproductive life, especially in adolescents younger than 15 and in women older than 35 or 40 years of age [15,25]. Regional studies report similar average ages, such as 26.7 years in Santander [13] and 28.1 years in Bolívar [21].

A recurring and highly relevant risk factor is affiliation with the subsidized health insurance scheme or a lack of health insurance coverage. In the Cali cohort, 65.7% belonged to these schemes (current study), which, although slightly lower than the national concentration reported in some reports, is consistent with the national finding of a higher risk of maternal mortality in these groups (RR 1.90 for subsidized, 1.57 for uninsured in 2022) [15]. This association underscores how maternal mortality is deeply linked to social and economic inequity, concentrating in populations with lower incomes and greater deprivation [5,19].

Likewise, the origin of patients from regions with greater vulnerabilities is another significant factor. In the Cali study, 20% originated from southwestern departments such as Cauca, Buenaventura, Nariño, and Chocó (current study), regions characterized by poverty, conflict, and access barriers [17]. This aligns with national evidence of a significantly higher risk for women from indigenous and Afro-Colombian ethnic groups (RR 4.93 and 3.34, respectively, in 2022) [15] and elevated MMRs in territories with a high population of these ethnicities [17,20]. The intersection of ethnicity, poverty, geographical location, and limited access to services generates multiple layers of vulnerability.

The identification of sickle cell anemia as a pre-existing condition in the Cali cohort (current study) is consistent with the Afro-descendant ethnic composition of the region, where this condition is more prevalent and significantly increases the risk of pregnancy complications.

Finally, the high proportion of early maternal deaths (85.7% within 42 days postpartum) in the Cali study (current study) contrasts with national figures (around 63%) [15] and other regional studies [21]. This could reflect the acute and rapidly fatal nature of complications in the cases selected for autopsy or differences in classification, suggesting the need for further investigation into the exact timing of these deaths in the region.

### 4.5. Significance of Pathological Findings

Clinical autopsy plays a fundamental role in maternal mortality audit and surveillance. It allows one to confirm or refute clinical diagnoses, identify unsuspected underlying pathologies (such as strongyloidiasis and miliary tuberculosis in the Cali study, or dengue in the Santander study), and precisely determine the final pathophysiological mechanisms that led to death. This detailed information is invaluable for understanding the causal chains of death and, potentially, identifying failures in care. In Colombia, protocols exist that indicate the performance of clinical necropsy in cases of maternal mortality where the cause is not clearly defined.

The discrepancy observed between the findings of the Cali autopsy series (with a predominance of indirect/infectious causes) and broader surveillance data (which often highlight direct causes like HDP and hemorrhage) could be explained, in part by the methodological difference. Autopsy studies, although susceptible to selection bias (e.g., overrepresentation of sudden deaths or medico-legal cases), provide a diagnosis considered the “gold standard.” This standard can reveal conditions that were clinically missed or misclassified on death certificates, which are often based on the final clinical impression without pathological confirmation [23,26,27]. Therefore, the higher proportion of certain infections (such as strongyloidiasis, tuberculosis, dengue) found in the autopsy series might reflect an underestimation of these causes in records based solely on clinical diagnoses, suggesting that the actual burden of infections as contributors to maternal mortality might be greater than indicated by general statistics.

The identification of specific infectious agents like strongyloidiasis and tuberculosis in Cali, and dengue in Santander, through autopsy highlights a critical intersection between prevalent endemic diseases in these regions and pregnancy. These are not the most commonly cited global causes of maternal mortality, but their emergence in local studies suggests that they are significant contributors in these particular contexts. It is plausible that pregnancy, due to its immunosuppressive physiological changes, exacerbates these infections, or that socioeconomic factors increase the exposure and vulnerability of pregnant women. This implies that maternal mortality prevention strategies must go beyond standard obstetric care and actively integrate the surveillance, diagnosis, and management of local endemic infections within maternal care protocols, requiring a geographically differentiated public health approach [2,8].

### 4.6. Study Limitations

It is relevant to consider certain inherent characteristics and limitations of this study when interpreting its results. Firstly, given its retrospective and descriptive design, the findings allow for characterizing the cases of maternal mortality verified by autopsy in this specific cohort but do not enable the establishment of definitive causal relationships or the evaluation of intervention effectiveness.

Likewise, the information available in autopsy records and retrospective clinical charts presented limited detailed data on prenatal care coverage and quality. While these factors are recognized as important determinants of maternal health at the population level, their detailed analysis was beyond the scope of the present study based on the data available for this cohort.

Finally, it is important to bear in mind that case selection was based on the performance of clinical autopsies, a procedure that is not applied to all maternal deaths. This nature of the sampling may introduce a potential for selection bias, as the autopsy cohort might consist of cases with particular characteristics (for example, those with less clinically clear causes of death or that meet medico-legal criteria). Therefore, the results of this study are representative of the population of women who died from maternal causes and who underwent autopsy at the reference hospital, and they complement the information derived from general epidemiological surveillance, without necessarily constituting a fully representative sample of the total maternal deaths in the region. Future research integrating diverse data sources could offer a broader perspective.

### 4.7. Future Recommendations

Based on the findings of this study and its comparison with the literature, the following lines of action and future research are proposed:

- Diagnostic Validation: Conduct prospective multicenter studies in Colombia that systematically compare clinical diagnoses of the cause of maternal mortality with autopsy findings to quantify concordance, identify the most frequent discrepancies, and evaluate the real added value of autopsy in epidemiological surveillance.

## 5. Conclusions

This retrospective autopsy study of maternal deaths at a referral hospital in Cali, the largest such series in Colombia, provides crucial pathological insights that complement epidemiological surveillance. It highlights a cohort characterized by significant socioeco-nomic vulnerability, including women affiliated with subsidized/uninsured health schemes and migrants from high-risk southwestern regions. Autopsy findings notably reveal a predominance of indirect and infectious causes (57.1%), often involving locally endemic diseases likely underestimated in clinically based data, while PPH remained the leading direct cause (50% of direct deaths).

They emphasize the profound impact of social inequities and regional disparities, demanding targeted clinical strategies (e.g., improved sepsis and endemic infection management, comprehensive sickle cell anemia care) and multisectoral public health interventions focused on addressing access barriers, enhancing the quality of care, and mitigating socioeconomic and geographic vulnerabilities for at-risk populations.

## Figures and Tables

**Table 1:** Sociodemographic and clinical characteristics.

Clinical and Sociodemographic Characteristics	Descriptor	Measure/Summary	
		N ()	%
Age (years)	15–20	10	23.8
	21–25	7	16.7
	26–30	11	26.2
	31–35	9	21.4
	36–40	5	11.9
Insurance Type	Private	6	14.3
	Public	26	61.6
	Uninsured	1	4.1
	No data	9	20
Number of Pregnancies	1	16	38.1
	2–4	19	5.2
	5–10	5	11.9
	More than 10	2	4.8
Gestational Weeks	6–12	4	9.5
	13–19	4	9.5
	20–27	10	23.8
	28–36	17	40.5
	37–43	7	16.7
History of Abortion	Yes	12	28.6
	No	30	71.4
Medical History	Sickle cell anemia	3	7.1
	Cirrhosis/atresia	1	2.4
	Malnutrition	2	4.8
	SLE (Systemic Lupus Eryth.)	3	7.1
	HTN (Hypertension)	3	7.1
	Obesity	1	2.4
	Nil of relevance	29	69
Type of Delivery	Vaginal	14	33.3
	Cesarean section	22	52.4
	Not specified	6	14.3
Level of Care	1	3	7.1
	2	3	7.1
	3	29	69
	4	7	16.7
Days between Admission and Death (Mean)	Direct death	4.2	
	Indirect death	16.5	
Type of Mortality	Early	36	85.7
	Late	6	14.3

N: number of cases; SLE: systemic lupus erythematosus; HTN: hypertension.

**Table 2:** Indirect causes of maternal mortality, 2000–2023.

Cause	n
Cardiogenic shock	
Hypertrophic cardiomyopathy secondary to mitral and aortic stenosis due to rheumatic fever	1
Severe mitral valve stenosis with left ventricular dilation	1
Distributive shock	
Bilateral basal pulmonary hemorrhage	1
Toxic hepatopathy	1
Sickle cell crisis	1
Metastatic dysgerminoma	1
Hypovolemic and septic shock	
Upper gastrointestinal bleeding, abdominal sepsis due to *Escherichia coli*	2
Autoimmune hemolytic anemia	1
Neurogenic shock	
Extensive ischemic cerebrovascular disease	1
Septic shock	
Sepsis—Bacteremia	4
Massive strongyloidiasis	2
Viral meningoencephalitis	1
Bacterial multilobar pneumonia	4
Cytomegalovirus pneumonia	1
Miliary tuberculosis	1
Pulmonary tuberculosis	1

**Table 3:** Direct/obstetric causes of maternal mortality, 2000–2023.

Cause	n
Cardiogenic/Distributive shock	
Amniotic fluid embolism	1
Hypovolemic shock	
Uterine hemorrhage	4
Hypertensive disorders associated with pregnancy	4
Neurogenic shock	
Fulminant hepatic failure secondary to acute fatty liver of pregnancy	1
Intracerebral hemorrhage due to arteriovenous malformation	1
Neurogenic and distributive shock	
Eclampsia with hemorrhagic stroke	1
Obstructive shock	
Pulmonary thromboembolism	3
Septic shock	
Acute panmetritis and salpingitis	1
Sepsis due to *Acinetobacter baumannii* secondary to septic abortion	1

**Table 4:** Comparison of key findings in maternal mortality studies in colombia.

Reference/ID	Location(s)	Study Period	Design/Type	Key Population/Sample	Reported MMR (Per 100 k LB)	Main Causes	Key Sociodemographic/Risk Factors
Current Study	Cali	2000–2023	Descriptive, observational, retrospective	42 autopsied maternal deaths	Not applicable (case-based, not population-based)	Indirect (57.1%) > Direct (42.9%). Main indirect: Septic shock (65.2%). Main direct: Hypovolemic shock due to postpartum hemorrhage (50%).	Public/Uninsured (65.7%). Age 26–30 (26.2%). From Cauca, Buenaventura, Nariño, Chocó (20% with migration history to Cali). Sickle cell anemia, SLE, nutritional disorders noted; 85.7% early deaths. Most received Level-3 or -4 care.
(Álvarez-Sierra, S.P., 2018) [23]	Santander	2012–2015	Descriptive, retrospective, cross-sectional	49 maternal deaths	Not specified in text	Hypertensive disorders, hemorrhagic complications, non-obstetric sepsis, obstetric sepsis, respiratory infections (per 2010–2012 data).	Mostly subsidized regime (57.1%), aged 22–35 (57.1%), mostly postpartum deaths (84%).
(Cárdenas-Cárdenas et al., 2015) [19]	Colombia (Municipal & Departmental)	2011	Ecological, analytical (two-level)	1094 municipalities (97.5% coverage)	69.3 (national)	Not detailed (focus on socioeconomic association).	Multidimensional poverty (municipal/departmental), low insurance (marginal), transparency index (departmental). Higher MMR in poorer departments.
(Ruíz et al., 2019) [13]	Santander (University Hospital)	2005–2018	Retrospective autopsy series	31 maternal deaths with autopsy	Not applicable	Indirect (67.8%) > Direct (32.2%). Infectious main among indirect (14 cases). Dengue most frequent (5 cases).	Average age 26.7, basic education, 67.8% died from indirect causes, 45.2% from infection.
(Bello-Muñoz et al., 2013) [21]	Bolívar	2010–2012	Descriptive retrospective (vital stats, SIVIGILA)	46 maternal deaths	46.6	Direct (62.7%) > Indirect (37.3%). Obstetric hemorrhage leading (MMR 11.1). Infections (MMR 9.12). Preeclampsia complications (MMR 8.1).	Mean age 28.1; 84.7% subsidized regime. Delay types I (73.9%) and IV (82.6%) common; 89.1% preventable deaths. Majority postpartum (58.6%).
(Vélez-Maya et al., 2019) [18]	Bogotá	2010–2012 and 2013–2015	Observational, retrospective, cross-sectional, analytical (sentinel cases)	225 early maternal deaths and 630,017 LB	39 (2010–2012) → 32 (2013–2015)	Direct (49.8%) > Indirect. Leading direct: Hypertensive disorders (16.8%), hemorrhage (13%), other obstetric complications (11%). Rise in infections and suicides (self-inflicted injuries).	Mostly postpartum. Age 25–29 highest proportion. Low education (basic), cohabiting; 96.4% not from special ethnic group. Subsidized and uninsured had higher mortality.
(Rodríguez Hernández et al., 2023) [17]	Chocó	2010–2018	Mixed (Ecological Analytical & Phenomenological)	131 deceased women, 58,352 LB	224	Causes not detailed; focus on system deficiencies and barriers.	Strong association with Multidimensional Poverty Index (MPI). Weak health infrastructure, geographical barriers, conflict, ethnicity, poverty. Role of traditional midwifery.
(Castañeda-Orjuela et al., 2023) [5]	Colombia (Municipal)	2019–2020	Time series, ecological	6342 deaths (2008–2020); subsample: 1055 (2019–2020)	80 (2019), 87 (2020)	Causes not detailed; focus on COVID-19 excess mortality and inequality.	Higher mortality in lowest socioeconomic-quintile municipalities (almost 3× higher). Inequality worsened in 2020. Barriers to essential service access.
(Mellizo, 2022) [15]	Colombia (National)	2022	Surveillance data analysis (SIVIGILA)	472 maternal deaths of residents	42.9 (Early maternal mortality)	Direct (60.9%) > Indirect (38.3%). Main direct: Hypertensive disorders (23.8%), hemorrhage (19.5%), pregnancy-related sepsis (7.7%). Main indirect: Other causes (13.4%).	Higher risk: >40 yrs (MMR 119.8). Subsidized (RR 1.90), Uninsured (RR 1.57). Indigenous (RR 4.93), Afro-Colombian (RR 3.34). Rural-dispersed (Ratio 72.0).
(Mosquera Córdoba & Cuesta Caicedo, 2022) [20]	Chocó	2013–2019	Descriptive retrospective (SIVIGILA forms)	96 reported maternal deaths	Increased from 108.8 (2009) to 152.2 (2019); peak 317.41 (2017)	Main cause: Hypertensive pregnancy disorders. 94% (68) direct obstetric causes vs. 6% (4) indirect.	Age groups 30–34 (17) and 20–24 (14) contributed most. 90% in SGSSS, 10% uninsured; 94% homemakers. Poor prenatal care by doctors (72.2%). Affected: Afro and Indigenous, rural.
(Vélez Cuervo et al., 2024) [22]	Antioquia	2012–2020	Descriptive retrospective	38 deaths from hypertensive pregnancy disorders	Not applicable (focused on hypertensive disorders)	THAE (eclampsia, HELLP, intracerebral hemorrhage, premature placental abruption).	Suboptimal care in 37/38 cases. Delayed detection, poor treatment (Mg sulfate, antihypertensives), lack of resources, administrative issues.
INS/SDS Bulletin 2024 [12]	Colombia (National)	2024 (PE VI preview)	Surveillance data analysis (SIVIGILA)	90 reported cases (PE VI 2024 preview)	45.0 (PE VI 2024 preview)	Direct (58%) > Indirect (33.3%) (Grouped causes). Main direct: Obstetric hemorrhage (12.2%), hypertensive disorders (18.9%).	Highest MMR in: Indigenous ethnicity (214.4), Small town residence (94.2), Uninsured (58.9), Age 10–14 (69.3) and >40 (138.0).

MMR: maternal mortality ratio; LB: live births; SLE: systemic lupus erythematosus.

## Data Availability

The original contributions presented in this study are included in the article. Further inquiries can be directed to the corresponding author.

## Abbreviations

The following abbreviations are used in this manuscript:

MM	Maternal Mortality
MMR	Maternal Mortality Ratio
LB	Live Births
WHO	World Health Organization
SLE	Systemic Lupus Erythematosus
HTN	Hypertension
N	Number of Cases
SLE	Systemic Lupus Erythematosus
PPH	Postpartum Hemorrhage
HDP	Hypertensive Disorders in Pregnancy
SCA	Sickle Cell Anemia
ANC	Antenatal Care
SMM	Severe Maternal Morbidity
PMR	Proportional Mortality Ratio
DANE	National Administrative Department of Statistics
SIVIGILA	National Public Health Surveillance System
